# Monosynaptic Input Mapping of Diencephalic Projections to the Cerebrospinal Fluid-Contacting Nucleus in the Rat

**DOI:** 10.3389/fnana.2020.00007

**Published:** 2020-02-28

**Authors:** Si-Yuan Song, Ying Li, Xiao-Meng Zhai, Yue-Hao Li, Cheng-Yi Bao, Cheng-Jing Shan, Jia Hong, Jun-Li Cao, Li-Cai Zhang

**Affiliations:** Jiangsu Province Key Laboratory of Anesthesiology, Xuzhou Medical University, Xuzhou, China

**Keywords:** CSF-contacting nucleus, diencephalon, thalamus, projection, retrograde trace

## Abstract

**Objective**: To investigate the projections the cerebrospinal fluid-contacting (CSF-contacting) nucleus receives from the diencephalon and to speculate on the functional significance of these connections.

**Methods**: The retrograde tracer cholera toxin B subunit (CB) was injected into the CSF-contacting nucleus in SD rats according to the experimental formula of the stereotaxic coordinates. Animals were perfused 7–10 days after the injection, and the diencephalon was sliced at 40 μm with a freezing microtome. CB-immunofluorescence was performed on all diencephalic sections. The features of CB-positive neuron distribution in the diencephalon were observed with a fluorescence microscope.

**Results**: The retrograde labeled CB-positive neurons were found in the epithalamus, subthalamus, and hypothalamus. Three functional diencephalic areas including 43 sub-regions revealed projections to the CSF-contacting nucleus. The CB-positive neurons were distributed in different density ranges: sparse, moderate, and dense.

**Conclusion**: Based on the connectivity patterns of the CSF-contacting nucleus that receives anatomical inputs from the diencephalon, we preliminarily assume that the CSF-contacting nucleus participates in homeostasis regulation, visceral activity, stress, emotion, pain and addiction, and sleeping and arousal. The present study firstly illustrates the broad projections of the CSF-contacting nucleus from the diencephalon, which implies the complicated functions of the nucleus especially for the unique roles of coordination in neural and body fluids regulations.

## Introduction

The cerebrospinal fluid (CSF)-contacting nucleus is a unique nucleus in the brain. It is located within the ventral gray of the lower portion of the aqueduct (Aq) and upper portion of the fourth ventricle (4V) floor (Song et al., 2019). The outstanding feature of this nucleus is that the neural somata are located in the brain parenchyma but the processes stretch into the CSF (Song and Zhang, 2018; Song et al., 2019). The morphological connections of the CSF-contacting nucleus with non-CSF-contacting neurons, glia cells, and blood vessels have been confirmed with electron microscopy (Zhang et al., 2003). The unique characteristic of the CSF-contacting nucleus implies that this nucleus may be a key structure bridging the nerve and fluids (CSF and plasma), and play an extremely important role in physiological activities. It has been approximately 30 years now since we first discovered, named and began to study this nucleus. The basic biological characteristics of the CSF-contacting nucleus, such as its specific labeling method (Lu et al., 2008), location and morphology, stereotaxic coordinates (Song et al., 2019), substance distributions [neurotransmitter (Lu et al., 2011), receptor (Liu P. F. et al., 2017), ion channels (Wang et al., 2014)] and its relationship with some biological activities [such as pain (Zhou et al., 2017), sodium appetite (Xing et al., 2015), stress (Wu et al., 2015), morphine dependence and withdrawal (Lu et al., 2011)] have been revealed. However, this nucleus is involved in pathways and mechanisms of different biological activities that are still to be clarified.

The diencephalon is located between the cerebral cortex and the mesencephalon and is covered by the cortex. It can be divided into five parts: dorsal thalamus, epithalamus, hypothalamus, metathalamus, and subthalamus. The dorsal thalamus serves as a relay center for the transmission of sensory and motor messages from the medulla oblongata and spinal cord to the cerebrum. The hypothalamus is a high center of visceral function modulation, and it is extremely important for the maintenance of homeostasis. The metathalamus processes the visual and auditory information to the cortex. The epithalamus (mainly habenula) is involved in reward processing and affective control (Hikosaka, 2010; Proulx et al., 2014). The subthalamus (zona incerta and subthalamic nucleus) participate in visceral, arousal and motor control (Mitrofanis, 2005; Telkes et al., 2018).

One of the aims of Neuroscience is to unveil the neural networks between different types of neurons to understand the functions of the brain (Watabe-Uchida et al., 2012). The unique feature of the CSF-contacting nucleus is to communicate with body fluids. The nucleus may form specific neural circuits with diencephalon in body fluids homeostasis and other functions. However, the connections between the diencephalon and the CSF-contacting nucleus have not been identified. To understand these connections and their possible biological significance, we injected retrograde tracer cholera toxin B subunit (CB) into the CSF-contacting nucleus. The projections of different diencephalic regions to the CSF-contacting nucleus can be revealed with immunofluorescence. A putative functional significance was speculated according to the projection relationships. Our study provides the first approach to further understand the biological significance of the diencephalic-CSF-contacting nucleus connections.

## Materials and Methods

### Experimental Animals

Specific pathogen-free (SPF) grade Sprague–Dawley rats (weight 250 ± 50 g) were acquired from the Experimental Animal Centre of Xuzhou Medical University. Rats successfully injected with the tracer into the CSF-contacting nucleus were used for observation and analysis (*n* = 6). All experiments were approved by the Committee for Ethical Use of Laboratory Animals, Xuzhou Medical University.

### Tracer Administration

Rats were anesthetized with pentobarbital sodium (40 mg/kg, i.p.), and heads were fixed on a stereotaxic instrument (Stoelting 51700, USA). A 1% CB solution (0.2 μl, Sigma, USA) was injected into the core of the CSF-contacting nucleus (Bregma: 8,242 ± 183 μm, Lateral: 92 ± 6 μm, Depth: 6,451 ± 109 μm; Song et al., 2019).

### Sampling and Histology

Seven to 10 days after the injection of the tracer, rats were perfused and sacrificed. Rats were anesthetized with pentobarbital sodium (40 mg/kg, i.p.) and perfused with 300 ml of phosphate-buffered saline (0.01 M PBS, pH 7.4), followed by 4% paraformaldehyde in 0.2 M phosphate buffer (300 ml, pH 7.4). The whole brain and spinal cord were isolated and sectioned coronally on a cryostat (Leica CM1900, Germany) at 40 μm. All sections were kept in sequence and numbered. In this study, only the diencephalon regions were captured and analyzed.

### Tracer Staining and Cell Counting

All sections were examined with CB immunofluorescence (rabbit anti-CB primary antibody diluted in 1:600, Abcam; donkey anti-rabbit Alexa Fluor 488 secondary antibody diluted in 1:200, Life Technologies, Carlsbad, CA, USA). Sections were mounted in sequence on slides, counterstained with DAPI and coverslipped. Diencephalon sections were imaged with a fluorescence microscope (Leica DM6, Germany) and a confocal laser microscope (Zeiss, Germany). The cell density of CB-positive neurons (cell number/0.2 mm^2^ area) in each brain region was calculated using Image-Pro Plus 7.0 software. The density of CB-positive neurons was classified as sparse, moderate and dense according to the densities: <5, 6–10 and >10, respectively.

### Three-Dimensional (3D) Reconstruction of Diencephalon Connections

The CB-positive neurons were aligned, segmented and registered according to the rat common reference atlas (Paxinos and Watson, 2007). The 3D diencephalon connections were reconstructed using the Imaris software, version 8.4.1 (Bitplane, USA). The “surface” module was used for rendering the brain regions in the diencephalon and the outline of the brain surface. The red areas represented strong connections; green areas represented moderate connections, and blue areas represented weak connections.

## Results

### Injection of the Retrograde Tracer Cholera Toxin B Subunit Into the CSF-Contacting Nucleus

**Figure 1:**
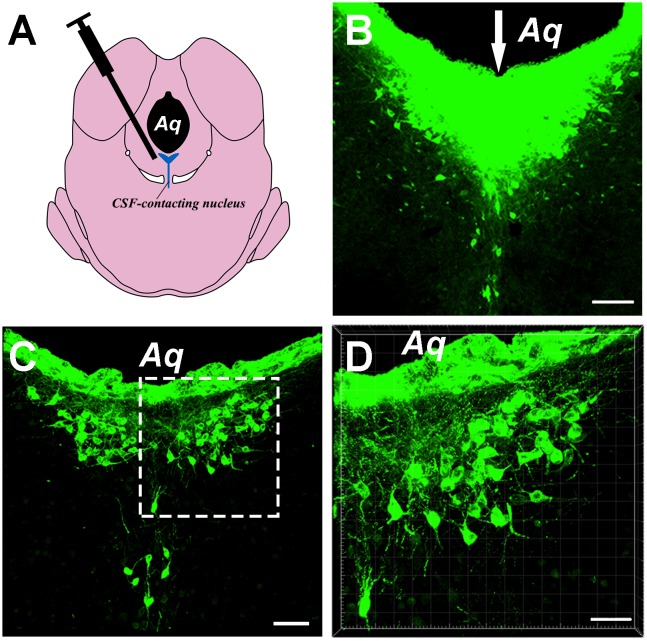
Image of the cholera toxin B subunit (CB)-tracer injection into the cerebrospinal fluid (CSF)-contacting nucleus. **(A)** The schematic diagram of the injection site to the CSF-contacting nucleus. **(B)** The tracer CB injection into the CSF-contacting nucleus. Green fluorescence labeling of the CB was seen in the entire CSF-contacting nucleus. The white arrow indicates the passage of the injected needle. **(C)** A representative section of the CSF-contacting nucleus in the brain. **(D)** Higher magnification of the boxed area in **(C)**. Aq, aqueduct. Scale bars: 100 μm in **(B)**; 70 μm in **(C)**; 40 μm in **(D)**.

Injections of the CB tracer produced dense positive staining (green). The tracer was confined within the boundary of the CSF-contacting nucleus, where the microsyringe needle tract can be seen to be located at the core of the CSF-contacting nucleus (Figures 1A,B). Representative sections of the CSF-contacting nucleus are shown in Figures 1C,D.

### Cellular Morphology of the Diencephalon Connections

After the retrograde tracer was injected into the CSF-contacting nucleus, it was transported retrogradely along the axons, and neuron somata projecting from the diencephalon was detected.

**Figure 2:**
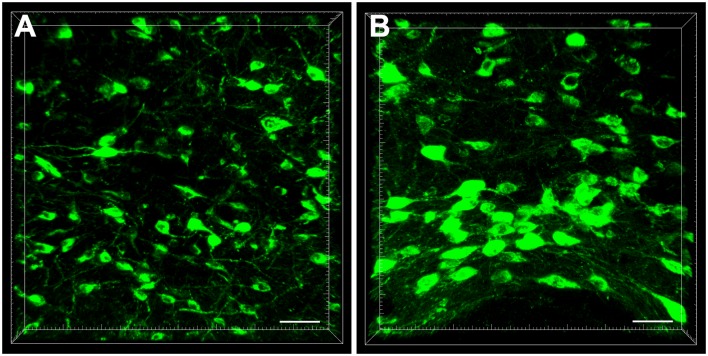
The cellular morphology of retrogradely labeled neurons in the diencephalon **(A,B)**. Scale bar: 40 μm.

In the diencephalon, the retrogradely labeled neurons appeared fusiform or polygon-shaped with different neuron sizes and clear processes. Some neurons were bipolar neurons with two obvious processes, while others were multipolar neurons with abundant dendrites (Figure 2).

### Connection Sites of the Diencephalon Regions

The entire diencephalon projections to the CSF-contacting nucleus could be identified by positive-labeled neurons and were mainly located in the hypothalamus, epithalamus, and subthalamus. Few or no positive-labeled neurons were identified in the dorsal thalamus and metathalamus.

**Figure 3:**
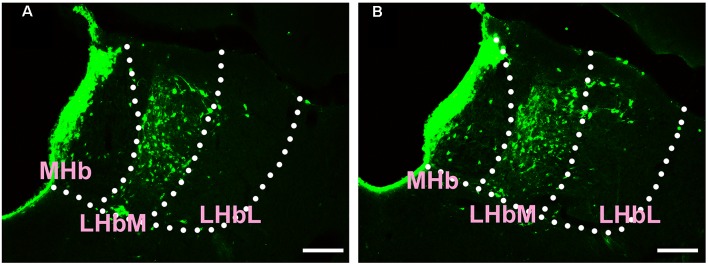
Distribution of CB-positive neurons in the epithalamus **(A,B)**. Abbreviations: MHb, medial habenular nucleus; LHbM, lateral habenular nucleus medial part; LHbL, lateral habenular nucleus lateral part. Scale bars: 100 μm.

In the epithalamus, CB-positive neurons were identified in the medial habenular nucleus (MHb), in the medial part of the lateral habenular nucleus (LHbM) and the lateral part of the lateral habenular nucleus (LHbL). Among them, the MHb and LHbM have strong connections, while the LHbL has sparse projections to the CSF-contacting nucleus (Figure 3).

**Figure 4:**
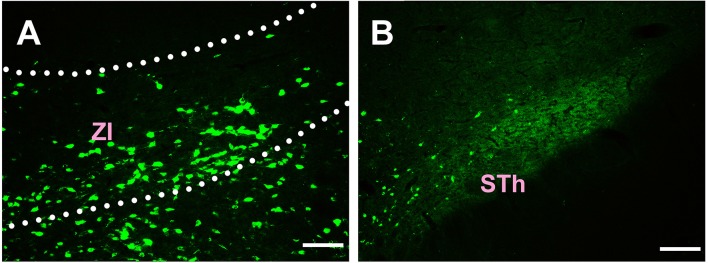
Distribution of CB-positive neurons in the subthalamus **(A,B)**. Abbreviations: ZI, zona incerta; STh, subthalamic nucleus. Scale bars: 100 μm.

In the subthalamus, CB-positive neurons were observed in the zona incerta (ZI) and subthalamic nucleus (STh). The ZI sends moderate projections, and STh sends sparse connections to the CSF-contacting nucleus (Figure 4).

**Figure 5:**
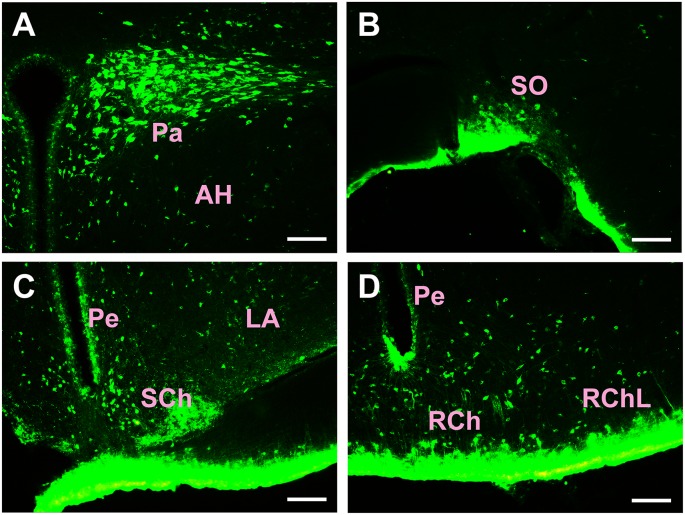
Distribution of CB-positive neurons in the hypothalamus Part I **(A–D)**. Abbreviations: Pa, paraventricular hypothalamic nucleus; AH, anterior hypothalamic area; SO, supraoptic nucleus; Pe, periventricular hypothalamic nucleus; SCh, suprachiasmatic nucleus; LA, lateroanterior hypothalamic nucleus; RCh, retrochiasmatic area; RChL, retrochiasmatic area lateral part. Scale bars: 100 μm.

**Figure 6:**
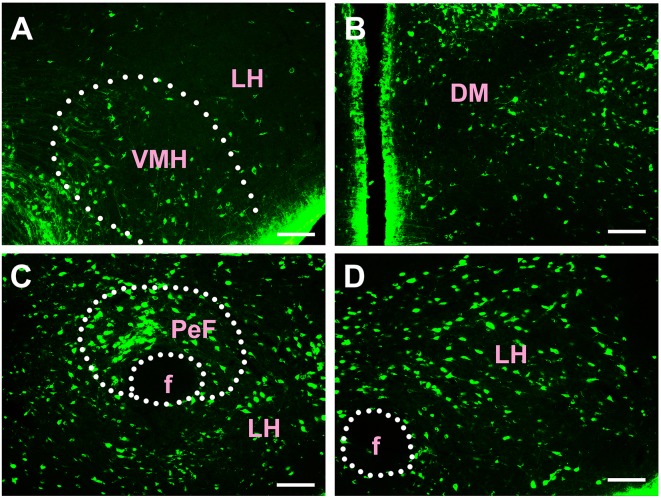
Distribution of CB-positive neurons in hypothalamus Part II **(A–D)**. VMH, ventromedial hypothalamic nucleus; LH, lateral hypothalamic area; DM, dorsomedial hypothalamic nucleus; PeF, perifornical nucleus; f, fornix. Scale bars: 100 μm.

**Figure 7:**
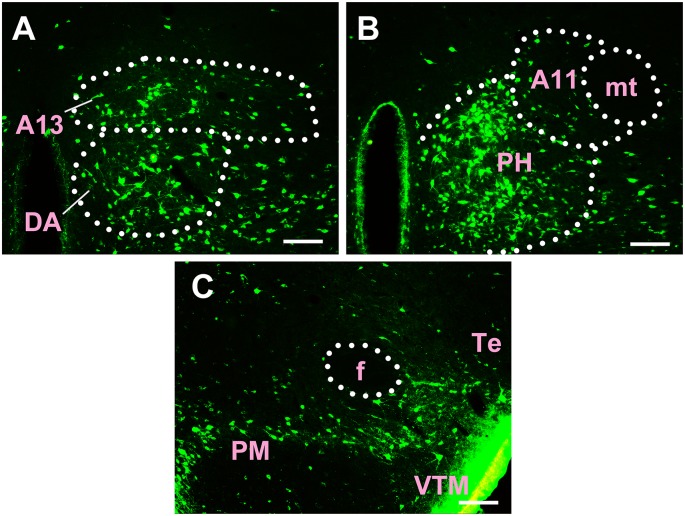
Distribution of CB-positive neurons in hypothalamus Part III **(A–C)**. A13, A13 dopamine cells; DA, dorsal hypothalamic area; A11, A11 dopamine cells; PH, posterior hypothalamic nucleus; mt, mammillothalamic tract; f, fornix; PM, premammillary nucleus; VTM, ventral tuberomammillary nucleus; Te, terete hypothalamic nucleus. Scale bars: 100 μm.

Most of the connections between the diencephalon and the CSF-contacting nucleus were observed in the hypothalamus. A total of 38 sub-regions in the hypothalamus formed projections of the CSF-contacting nucleus. These include: the paraventricular hypothalamic nucleus (Pa), periventricular hypothalamic nucleus (Pe), anterior hypothalamic area (AH), lateroanterior hypothalamic nucleus (LA), suprachiasmatic nucleus (SCh), supraoptic nucleus (SO), supraoptic nucleus, retrochiasmatic part (SOR), episupraoptic nucleus (ESO), subparaventricular zone of the hypothalamus (SPa), retrochiasmatic area (RCh), retrochiasmatic area lateral part (RChL), lateral hypothalamic area (LH), accessory neurosecretory nuclei (ANS), dorsal hypothalamic area (DA), stigmoid hypothalamic nucleus (Stg), arcuate hypothalamic nucleus (Arc), dorsomedial hypothalamic nucleus (DM), ventromedial hypothalamic nucleus (VMH), A11 dopamine cells (A11), A13 dopamine cells (A13), medial tuberal nucleus (MTu), terete hypothalamic nucleus (Te), paraterete nucleus (PTe), perifornical nucleus (PeF), subincertal nucleus (SubI), posterior hypothalamic nucleus (PH), dorsal part of the posterior hypothalamic area (PHD), ventral part of the pre-mammillary nucleus (PMV), dorsal part of the pre-mammillary nucleus (PMD), dorsal tuberomammillary nucleus (DTM), ventral tuberomammillary nucleus (VTM), lateral mammillary nucleus (LM), parasubthalamic nucleus (PSTh), gemini hypothalamic nucleus (Gem), sub-mammillothalamic nucleus (SMT), prerubral field (PR), rostral interstitial nucleus of medial longitudinal fasciculus (RI), and fields of Forel (F; Figures 5–7).

Among the hypothalamic regions, the Pa, AH, LA, SCh, SO, RCh, RChL, LH, ANS, DA, STg, Arc, DM, VMH, A11, A13, MTu, Te, PeF, SubI, PH, PHD, PMV, PMD, DTM, and LM send strong projections to the CSF-contacting nucleus; the SOR, SPa, PTe, VTM, PSTh, Gem, and SMT send moderate projections; the Pe, ESO, PR, RI, and F send sparse connections to the CSF-contacting nucleus (Figures 5–7).

In summary, CB-positive neurons were distributed in three functional areas including 43 sub-regions in the diencephalon and ranged from sparse, moderate and dense. CB-positive neurons were mainly located in the epithalamus, subthalamus, and hypothalamus. The dorsal thalamus and metathalamus did not contain CB-positive neurons.

### 3D Reconstruction of CB-Positive Diencephalic Neurons

**Figure 8:**
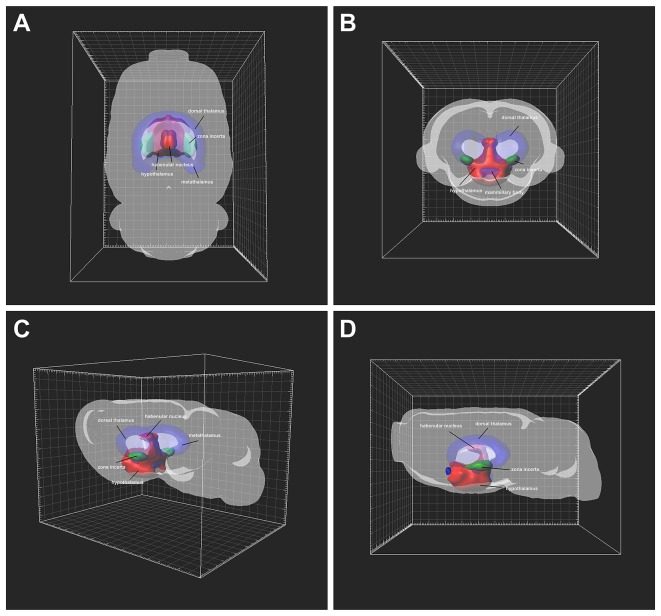
Three-Dimensional (3D) view of the diencephalon connection patterns to the CSF-contacting nucleus **(A–D)**. Red areas are strong connections; green areas are moderate connections; blue areas are weak connections.

The distribution of CB-positive neurons throughout the diencephalon was 3D reconstructed. The density of the connections became clear in a 3D view. Red areas were dense connections (MHb, LHbM, Pa, AH, LA, SCh, SO, RCh, RChL, LH, ANS, DA, Stg, Arc, DM, VMH, A11, A13, MTu, Te, PeF, SubI, PH, PHD, PMV, PMD, DTM, and LM); green areas were moderate connections (ZI, SOR, SPa, PTe, VTM, PSTh, Gem, and SMT); and blue areas were sparse connections (LHbL, STh, Pe, ESO, PR, RI, and F; Figure 8).

### Number of Projections From Diencephalic Regions to the CSF-Contacting Nucleus

**Figure 9:**
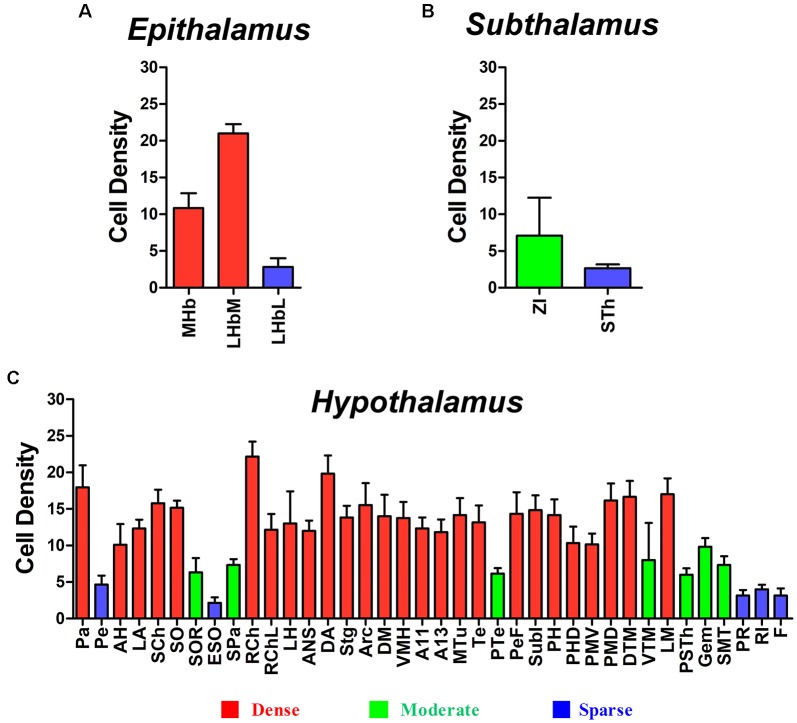
Diencephalic CB-positive neuronal input to the CSF-contacting nucleus (mean ± SD, *n* = 6). **(A)** Epithalamus; **(B)** Subthalamus; and **(C)** Hypothalamus.

In the diencephalon, the CB positive neurons were found in three functional areas including 43 sub-regions. The number of projections from these regions to the CSF- contacting nucleus is shown in Figure 9.

## Discussion

The CSF-contacting nucleus is a unique nucleus in the brain. This nucleus has non-synaptic connections between the CSF-contacting neurons and blood vessels and the CSF, and plays an important role in the regulation of body fluids; it has also synaptic connections between the CSF-contacting and non-CSF-contacting neurons, and it carries out nervous crosstalk in the brain. The unique anatomical features of the CSF-contacting nucleus imply that it may be a key structure bridging the nervous-and humor- regulating systems. The connections between the CSF-contacting neurons and blood vessels and CSF have been described previously (Zhang et al., 2003). The projections that this nucleus receives from the central nervous system have not been described in detail, although the bidirectional synapses between CSF-contacting and non-CSF-contacting neurons have been previously observed in the parenchyma with electron microscopy (Liang et al., 2007). This study provides a systematic report of the projections the CSF-contacting nucleus receives from different diencephalic functional regions.

**Figure 10:**
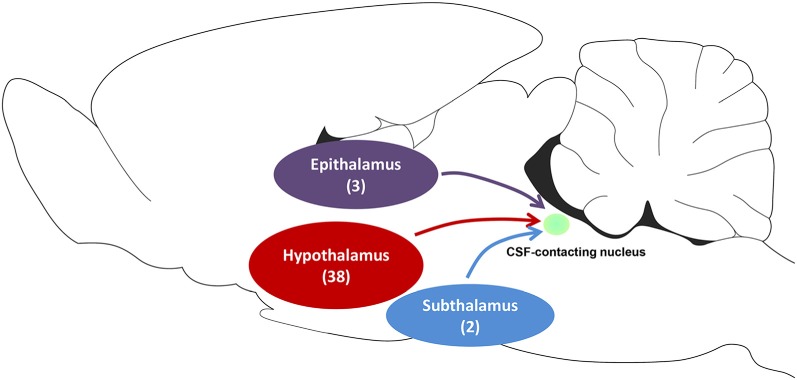
The schematic diagram of projections from functional areas in the diencephalon to the CSF-contacting nucleus. Among them, epithalamus contains three sub-regions, the hypothalamus contains 38 sub-regions, and subthalamus contains two sub-regions.

Our results indicate that the CSF-contacting nucleus receives extensive projections from three functional areas including 43 sub-regions of the diencephalon (Figure 10), which form the focus of several basic and clinical studies. Taking into account that some of these diencephalic functions have been described previously, the biological functions of the CSF-contacting nucleus may be predicted based on its connection patterns.

### Functional Implications

#### Homeostasis Regulation

The CSF-contacting nucleus receives dense projections from the hypothalamus, which might participate in homeostasis modulation. The role of homeostasis, modulated by the nervous, fluid, and immune systems, is to maintain the coordinated function of organs and systems in life activities, and a healthy physiological equilibrium in a changing world (Burdakov, 2019). The hypothalamus is a key structure for homeostasis regulation such as ambient temperature, energy balance, water-electrolyte metabolism, and biological rhythm.

##### Energy Balance

It is well established that hypothalamic regions play an important role in the regulation of feeding behavior, which contributes to processes of energy homeostasis (Flier and Maratos-Flier, 1998; Elmquist et al., 1999). The relevant hypothalamic nuclei engaged in this function, including the Arc, DM, VMH, LH, Pa, and PM, have extensive projections to the CSF-contacting nucleus. Disruption of the VMH functions causes obesity, while disruption of the LH functions induces weight loss, suggesting the VMH as a “satiety” and the LH as a “feeding” center (Hoebel and Teitelbaum, 1962). The Arc nucleus is extremely important for appetite and energy expenditure (Wei et al., 2018). Other hypothalamic nuclei are also crucial for the regulation of energy balance (Denroche et al., 2016; Péterfi et al., 2018; Zhang et al., 2018).

##### Fluids

Hypothalamic nuclei, such as the SO, LH and PSTh, can modulate the fluid homeostasis and project extensively to the CSF-contacting nucleus. The SO can sense the plasma osmolality and stabilize it *via* the release of arginine vasopressin (AVP; Prager-Khoutorsky and Bourque, 2015; Sandgren et al., 2018). The LH is known for its essential role in regulating the drinking behavior to maintain the homeostasis of fluids (Kurt et al., 2019). The PSTh is anatomically linked to parabrachial regions and participates in regulating the appetite for salt (Shin et al., 2011).

##### Biological Rhythm

The hypothalamic SCh and SPa have connections to the CSF-contacting nucleus and can both modulate the biological rhythm. The SCh is known as a master brain clock controlling the circadian rhythms. Body activities synchronize the metabolism, cognition and various behaviors to the environmental day-night cycle (Mohawk et al., 2012). The SPa is also involved in circadian rhythm regulation. It is a hub structure that relays the circadian information from the SCh to other brain areas and finally controls the circadian rhythms of various physiological processes (Lu et al., 2001). Recently, the LHb in the epithalamus was confirmed to express clock genes involved in the regulation of circadian functions (Salaberry et al., 2019).

#### Visceral Activity

The hypothalamus is a higher center of visceral activity modulation. Activation of the hypothalamus can produce significant autonomic responses. Among the hypothalamic regions and nuclei that mediate the visceral activity regulation are the Pa, DM, VMH, VTM, PeF, PH and PSTh, which project extensively to the CSF-contacting nucleus. The Pa is regarded as an integrative region that modulates the sympathetic outflow and cardiovascular activity (Coote, 2005; Li et al., 2019). Electrical stimulation of the DM and PeF results in tachycardia in rats (López-Gonzalez et al., 2013). Lesions in the VMH can aggravate the gastric mucosal injury through the vagal nerve pathway (Sun H. et al., 2018). The VTM is a nucleus with a high density of histaminergic neurons and is involved in arterial pressure control. Electrical or chemical stimulation of the PH increases arterial pressure (Yamanaka et al., 2017), heart rate, and sympathetic nerve activity (Gao et al., 2016). The PSTh also participates in cardiovascular regulation (Ciriello et al., 2008).

#### Stress

The CSF-contacting nucleus receives input from hypothalamic Pa, SO and DA and may participate in stress. Stress responses activate the hypothalamic-pituitary-adrenal (HPA) axis, where Pa can release hormones, such as the corticotropin-releasing hormone (CRH) and AVP, that affect biological activities (Joseph and Whirledge, 2017). The SO also expresses AVP as well as other substances, such as oxytocin, in response to a wide variety of stressors (Neumann, 2007; Borrow et al., 2018). In rats, the DA neurons are activated during stress as illustrated by c-Fos expression (Sarkar et al., 2007), and DA neurons are involved in mediating stress-induced hyperthermia (Machado et al., 2018).

#### Emotion

Both the LHb and MHb in the epithalamus have strong projections to the CSF-contacting nucleus. Lesion and genetic studies in mice and zebrafish respectively showed neuron burst firing under depression, which can be significantly reversed with antidepressants (Kim et al., 2018). Deep brain stimulation of the STh in patients with Parkinson’s disease, improves anxiety and mood disorders (Eisenstein et al., 2014; Gourisankar et al., 2018). The hypothalamic VMH is involved in anxiety and is a target of anxiolytic substances (Jiang et al., 2018). Moreover, the DM regulates panic-related defensive behavior and produces escape behavior (de Bortoli et al., 2013).

#### Pain and Addiction

The CSF-contacting nucleus receives input from the Pa, SO, A11, ZI, and STh and may participate in pain modulation. The Pa is involved in visceral hypersensitivity as revealed by colorectal distension (CRD; Zhang et al., 2016; Tang et al., 2017). The Pa and SO synthesize and release oxytocin, which participates in neuropathic pain (Sun W. et al., 2018). The A11 hypothalamic nucleus regulates the trigeminal analgesia and migraine headache (Kagan et al., 2013; Abdallah et al., 2015). The ZI and STh are subthalamus structures. Many studies have confirmed the role of ZI in pain processing *via* different nociceptive pathways and various mechanisms (Trageser and Keller, 2004; Cavdar et al., 2006; Masri et al., 2009; Moon et al., 2016). The deep brain stimulation of the STh can produce significant improvement of overall pain in patients with advanced Parkinson’s disease (Oshima et al., 2012).

The epithalamus (MHb and LHb) and LH in the hypothalamus project to the CSF-contacting nucleus and may participate in drug addiction. The MHb plays a significant role in drug addiction, especially nicotine and opioid addiction because it contains a high density of nicotinic acetylcholine receptors and μ opioid receptors (Fowler and Kenny, 2014; Gardon et al., 2014; Shih et al., 2014). However, the LHb correlates with the negative affective state after withdrawal from drug abuse (Mathis and Kenny, 2019). The LH is involved in drug-seeking behavior elicited by drug-associated stimuli.

#### Sleeping and Arousal

The CSF-contacting nucleus receives input from the LH, PeF, and ZI and may participate in sleeping and arousal. Different neuron types in the tuberomammillary nucleus and LH are implicated in the sleep/wakefulness regulation (Saito et al., 2018). The PeF receives inputs from circadian rhythm messages of the SCh to promote waking (Zhong et al., 2017). The stimulation of thalamic-ZI projections induce a sleep-like state (Liu et al., 2015), and a GABAergic subpopulation of neurons in the ZI can promote sleep (Liu K. et al., 2017).

In this study, we used a tract-tracing method to reveal the CSF-contacting nucleus input patterns from the diencephalon. The unique morphological feature of the CSF-contacting nucleus is that the somata are located in the brain parenchyma and can receive input from the above diencephalon areas; the processes can form synaptic and non-synaptic connections with non-CSF-contacting neurons, CSF, or plasma. Circuits forming between the diencephalon→CSF-contacting nucleus→non-CSF-contacting neurons may participate in the regulation of life activities *via* neuron-neuron crosstalk. Circuits that involve diencephalon→CSF-contacting nucleus→CSF/plasma may modulate physiological functions *via* neuron-fluid interactions. Based on the connections between the CSF-contacting nucleus and the diencephalon, we propose that the CSF-contacting nucleus participates in homeostasis regulation, visceral activity, stress, emotion, pain and addiction, sleeping and arousal, among others. Our study provides morphological evidence for further unveiling the significance of CSF-contacting nucleus in brain functions.

## Data Availability Statement

The data that support the findings of this study are available from the corresponding author, upon reasonable request.

## Ethics Statement

All animal experiments were approved by and performed in accordance with the guidelines of the Committee for Ethical Use of Laboratory Animals of Xuzhou Medical University.

## Author Contributions

S-YS and L-CZ designed the study and prepared the manuscript. S-YS, YL, X-MZ, Y-HL, C-YB, C-JS, JH, and J-LC conducted the studies. All authors read and approved the manuscript.

## Conflict of Interest

The authors declare that the research was conducted in the absence of any commercial or financial relationships that could be construed as a potential conflict of interest.

## References

[B1] Abdallah K. Monconduit L. Artola A. Luccarini P. Dallel R. (2015). GABAAergic inhibition or dopamine denervation of the A11 hypothalamic nucleus induces trigeminal analgesia. Pain 156, 644–655. 10.1097/j.pain.0000000000000091 25790455

[B2] Borrow A. P. Bales N. J. Stover S. A. Handa R. J. (2018). Chronic variable stress induces sex-specific alterations in social behavior and neuropeptide expression in the mouse. Endocrinology 159, 2803–2814. 10.1210/en.2018-00217 29788320 PMC6692887

[B3] Burdakov D. (2019). Reactive and predictive homeostasis: roles of orexin/hypocretin neurons. Neuropharmacology 154, 61–67. 10.1016/j.neuropharm.2018.10.024 30347195

[B4] Cavdar S. Onat F. Cakmak Y. O. Saka E. Yananli H. R. Aker R. (2006). Connections of the zona incerta to the reticular nucleus of the thalamus in the rat. J. Anat. 209, 251–258. 10.1111/j.1469-7580.2006.00600.x 16879603 PMC2100320

[B5] Ciriello J. Solano-Flores L. P. Rosas-Arellano M. P. Kirouac G. J. Babic T. (2008). Medullary pathways mediating the parasubthalamic nucleus depressor response. Am. J. Physiol. Regul. Integr. Comp. Physiol. 294, R1276–R1284. 10.1152/ajpregu.00437.2007 18287224

[B6] Coote J. H. (2005). A role for the paraventricular nucleus of the hypothalamus in the autonomic control of heart and kidney. Exp. Physiol. 90, 169–173. 10.1113/expphysiol.2004.029041 15604110

[B7] de Bortoli V. C. Yamashita P. S. Zangrossi H. Jr. (2013). 5-HT1A and 5-HT2A receptor control of a panic-like defensive response in the rat dorsomedial hypothalamic nucleus. J. Psychopharmacol. Oxford 27, 1116–1123. 10.1177/0269881113492900 23787365

[B8] Denroche H. C. Glavas M. M. Tuduri E. Karunakaran S. Quong W. L. Philippe M.. (2016). Disrupted leptin signaling in the lateral hypothalamus and ventral premammillary nucleus alters insulin and glucagon secretion and protects against diet-induced obesity. Endocrinology 157, 2671–2685. 10.1210/en.2015-1998 27183315

[B9] Eisenstein S. A. Dewispelaere W. B. Campbell M. C. Lugar H. M. Perlmutter J. S. Black K. J.. (2014). Acute changes in mood induced by subthalamic deep brain stimulation in Parkinson disease are modulated by psychiatric diagnosis. Brain Stimul. 7, 701–708. 10.1016/j.brs.2014.06.002 25017671 PMC4167923

[B10] Elmquist J. K. Elias C. F. Saper C. B. (1999). From lesions to leptin : hypothalamic control of food intake and body weight. Neuron 22, 221–232. 10.1016/s0896-6273(00)81084-3 10069329

[B11] Flier J. S. Maratos-Flier E. (1998). Obesity and the hypothalamus: novel peptides for new pathways. Cell 92, 437–440. 10.1016/s0092-8674(00)80937-x 9491885

[B12] Fowler C. D. Kenny P. J. (2014). Nicotine aversion: neurobiological mechanisms and relevance to tobacco dependence vulnerability. Neuropharmacology 76, 533–544. 10.1016/j.neuropharm.2013.09.008 24055497 PMC3858456

[B13] Gao H. R. Zhuang Q. X. Li B. Li H. Z. Chen Z. P. Wang J. J.. (2016). Corticotropin releasing factor excites neurons of posterior hypothalamic nucleus to produce tachycardia in rats. Sci. Rep. 6:20206. 10.1038/srep20206 26831220 PMC4735335

[B14] Gardon O. Faget L. Chu Sin Chung P. Matifas A. Massotte D. Kieffer B. L. (2014). Expression of mu opioid receptor in dorsal diencephalic conduction system: new insights for the medial habenula. Neuroscience 277, 595–609. 10.1016/j.neuroscience.2014.07.053 25086313 PMC4164589

[B15] Gourisankar A. Eisenstein S. A. Trapp N. T. Koller J. M. Campbell M. C. Ushe M.. (2018). Mapping movement, mood, motivation and mentation in the subthalamic nucleus. R. Soc. Open Sci. 5:171177. 10.1098/rsos.171177 30109035 PMC6083651

[B16] Hikosaka O. (2010). The habenula: from stress evasion to value-based decision-making. Nat. Rev. Neurosci. 11, 503–513. 10.1038/nrn2866 20559337 PMC3447364

[B17] Hoebel B. G. Teitelbaum P. (1962). Hypothalamic control of feeding and self-stimulation. Science 135, 375–377. 10.1126/science.135.3501.375 13907995

[B18] Jiang J. H. Peng Y. L. Zhang P. J. Xue H. X. He Z. Liang X. Y.. (2018). The ventromedial hypothalamic nucleus plays an important role in anxiolytic-like effect of neuropeptide S. Neuropeptides 67, 36–44. 10.1016/j.npep.2017.11.004 29195839

[B19] Joseph D. N. Whirledge S. (2017). Stress and the HPA axis: balancing homeostasis and fertility. Int. J. Mol. Sci. 18:E2224. 10.3390/ijms18102224 29064426 PMC5666903

[B20] Kagan R. Kainz V. Burstein R. Noseda R. (2013). Hypothalamic and basal ganglia projections to the posterior thalamus: possible role in modulation of migraine headache and photophobia. Neuroscience 248, 359–368. 10.1016/j.neuroscience.2013.06.014 23806720 PMC3858508

[B21] Kim D. Cheong E. Shin H. S. (2018). Overcoming depression by inhibition of neural burst firing. Neuron 98, 878–879. 10.1016/j.neuron.2018.05.032 29879390

[B22] Kurt G. Woodworth H. L. Fowler S. Bugescu R. Leinninger G. M. (2019). Activation of lateral hypothalamic area neurotensin-expressing neurons promotes drinking. Neuropharmacology 154, 13–21. 10.1016/j.neuropharm.2018.09.038 30266601 PMC6433557

[B23] Li P. Jie Y. YuGen S. Yu W. Yan S. (2019). High mobility group box-1 in hypothalamic paraventricular nuclei attenuates sympathetic tone in rats at post-myocardial infarction. Cardiol. J. 26, 555–563. 10.5603/CJ.a2018.0117 30338842 PMC8084387

[B24] Liang D. Zhang L. C. Qin C. Zeng Y. M. (2007). The ultrastructure of the distal CSF-contacting neurons in the dorsal raphe and the relationships with their surrounding tissues. Acta Anat. Sin. 38, 410–413. 10.3321/j.issn:0529-1356.2007.04.007

[B27] Liu P. F. Fang H. Z. Yang Y. Zhang Q. Q. Zhou Q. Q. Chen S. S.. (2017). Activation of P2X3 receptors in the cerebrospinal fluid-contacting nucleus neurons reduces formalin-induced pain behavior *via* PAG in a rat model. Neuroscience 358, 93–102. 10.1016/j.neuroscience.2017.06.036 28673711

[B26] Liu K. Kim J. Kim D. W. Zhang Y. S. Bao H. Denaxa M.. (2017). Lhx6-positive GABA-releasing neurons of the zona incerta promote sleep. Nature 548, 582–587. 10.1038/nature23663 28847002 PMC5958617

[B25] Liu J. Lee H. J. Weitz A. J. Fang Z. Lin P. Choy M.. (2015). Frequency-selective control of cortical and subcortical networks by central thalamus. Elife 4:e09215. 10.7554/elife.09215 26652162 PMC4721962

[B28] López-Gonzalez M. V. Díaz-Casares A. Peinado-Aragonés C. A. Lara J. P. Barbancho M. A. Dawid-Milner M. S. (2013). Neurons of the A5 region are required for the tachycardia evoked by electrical stimulation of the hypothalamic defence area in anaesthetized rats. Exp. Physiol. 98, 1279–1294. 10.1113/expphysiol.2013.072538 23525246

[B30] Lu X. Geng X. Zhang L. C. Zeng Y. (2008). The methodology for labeling the distal cerebrospinal fluid-contacting neurons in rats. J. Neurosci. Methods 168, 98–103. 10.1016/j.jneumeth.2007.09.033 18179825

[B31] Lu X. F. Li Y. Y. Wang C. G. Wei J. Q. Ye Y. Zhang L. C.. (2011). Substance P in the cerebrospinal fluid-contacting nucleus contributes to morphine physical dependence in rats. Neurosci. Lett. 488, 188–192. 10.1016/j.neulet.2010.11.026 21093542

[B29] Lu J. Zhang Y. H. Chou T. C. Gaus S. E. Elmquist J. K. Shiromani P.. (2001). Contrasting effects of ibotenate lesions of the paraventricular nucleus and subparaventricular zone on sleep-wake cycle and temperature regulation. J. Neurosci. 21, 4864–4874. 10.1523/jneurosci.21-13-04864.2001 11425913 PMC3508730

[B38] Machado N. L. S. Abbott S. B. G. Resch J. M. Zhu L. Arrigoni E. Lowell B. B.. (2018). A glutamatergic hypothalamomedullary circuit mediates thermogenesis, but not heat conservation, during stress-induced hyperthermia. Curr. Biol. 28, 2291.e5–2301.e5. 10.1016/j.cub.2018.05.064 30017482 PMC6085892

[B32] Masri R. Quiton R. L. Lucas J. M. Murray P. D. Thompson S. M. Keller A. (2009). Zona incerta: a role in central pain. J. Neurophysiol. 102, 181–191. 10.1152/jn.00152.2009 19403748 PMC2712264

[B33] Mathis V. Kenny P. J. (2019). From controlled to compulsive drug-taking: the role of the habenula in addiction. Neurosci. Biobehav. Rev. 106, 102–111. 10.1016/j.neubiorev.2018.06.018 29936111 PMC9871871

[B34] Mitrofanis J. (2005). Some certainty for the “zone of uncertainty”? Exploring the function of the zona incerta. Neuroscience 130, 1–15. 10.1016/j.neuroscience.2004.08.017 15561420

[B35] Mohawk J. A. Green C. B. Takahashi J. S. (2012). Central and peripheral circadian clocks in mammals. Annu. Rev. Neurosci. 35, 445–462. 10.1146/annurev-neuro-060909-153128 22483041 PMC3710582

[B36] Moon H. C. Lee Y. J. Cho C. B. Park Y. S. (2016). Suppressed GABAergic signaling in the zona incerta causes neuropathic pain in a thoracic hemisection spinal cord injury rat model. Neurosci. Lett. 632, 55–61. 10.1016/j.neulet.2016.08.035 27561604

[B37] Neumann I. D. (2007). Stimuli and consequences of dendritic release of oxytocin within the brain. Biochem. Soc. Trans. 35, 1252–1257. 10.1042/bst0351252 17956324

[B39] Oshima H. Katayama Y. Morishita T. Sumi K. Otaka T. Kobayashi K.. (2012). Subthalamic nucleus stimulation for attenuation of pain related to Parkinson disease. J. Neurosurg. 116, 99–106. 10.3171/2011.7.JNS11158 21905799

[B40] Paxinos G. Watson C. (2007). The Rat Brain in Stereotaxic Coordinates. 6th Edn. Amsterdam; Boston: Academic Press/Elsevier.

[B41] Péterfi Z. Farkas I. Denis R. G. P. Farkas E. Uchigashima M. Füzesi T.. (2018). Endocannabinoid and nitric oxide systems of the hypothalamic paraventricular nucleus mediate effects of NPY on energy expenditure. Mol. Metab. 18, 120–133. 10.1016/j.molmet.2018.08.007 30274714 PMC6308028

[B42] Prager-Khoutorsky M. Bourque C. W. (2015). Mechanical basis of osmosensory transduction in magnocellular neurosecretory neurones of the rat supraoptic nucleus. J. Neuroendocrinol. 27, 507–515. 10.1111/jne.12270 25712904

[B43] Proulx C. D. Hikosaka O. Malinow R. (2014). Reward processing by the lateral habenula in normal and depressive behaviors. Nat. Neurosci. 17, 1146–1152. 10.1038/nn.3779 25157511 PMC4305435

[B44] Saito Y. C. Maejima T. Nishitani M. Hasegawa E. Yanagawa Y. Mieda M.. (2018). Monoamines inhibit GABAergic neurons in ventrolateral preoptic area that make direct synaptic connections to hypothalamic arousal neurons. J. Neurosci. 38, 6366–6378. 10.1523/jneurosci.2835-17.2018 29915137 PMC6596100

[B45] Salaberry N. L. Hamm H. Felder-Schmittbuhl M. P. Mendoza J. (2019). A suprachiasmatic-independent circadian clock(s) in the habenula is affected by Per gene mutations and housing light conditions in mice. Brain Struct. Funct. 224, 19–31. 10.1007/s00429-018-1756-4 30242505

[B46] Sandgren J. A. Linggonegoro D. W. Zhang S. Y. Sapouckey S. A. Claflin K. E. Pearson N. A.. (2018). Angiotensin AT1A receptors expressed in vasopressin-producing cells of the supraoptic nucleus contribute to osmotic control of vasopressin. Am. J. Physiol. Regul. Integr. Comp. Physiol. 314, R770–R780. 10.1152/ajpregu.00435.2017 29364700 PMC6032302

[B47] Sarkar S. Zaretskaia M. V. Zaretsky D. V. Moreno M. DiMicco J. A. (2007). Stress- and lipopolysaccharide-induced c-fos expression and nNOS in hypothalamic neurons projecting to medullary raphe in rats: a triple immunofluorescent labeling study. Eur. J. Neurosci. 26, 2228–2238. 10.1111/j.1460-9568.2007.05843.x 17927775

[B48] Shih P. Y. Engle S. E. Oh G. Deshpande P. Puskar N. L. Lester H. A.. (2014). Differential expression and function of nicotinic acetylcholine receptors in subdivisions of medial habenula. J. Neurosci. 34, 9789–9802. 10.1523/jneurosci.0476-14.2014 25031416 PMC4099552

[B49] Shin J. W. Geerling J. C. Stein M. K. Miller R. L. Loewy A. D. (2011). FoxP2 brainstem neurons project to sodium appetite regulatory sites. J. Chem. Neuroanat. 42, 1–23. 10.1016/j.jchemneu.2011.05.003 21605659 PMC3148274

[B51] Song S. Y. Li Y. H. Bao C. Y. Li Y. Yin P. C. Hong J.. (2019). Stereotaxic coordinates and morphological characterization of a unique nucleus (CSF-contacting nucleus) in rat. Front. Neuroanat. 13:47. 10.3389/fnana.2019.00047 31143102 PMC6520827

[B50] Song S. Y. Zhang L. C. (2018). The establishment of a CSF-contacting nucleus “knockout” model animal. Front. Neuroanat. 12:22. 10.3389/fnana.2018.00022 29636668 PMC5881085

[B52] Sun H. Zhao P. Liu W. Li L. Ai H. Ma X. (2018). Ventromedial hypothalamic nucleus in regulation of stress-induced gastric mucosal injury in rats. Sci. Rep. 8:10170. 10.1038/s41598-018-28456-0 29977067 PMC6033936

[B53] Sun W. Zhou Q. Ba X. Feng X. Hu X. Cheng X.. (2018). Oxytocin relieves neuropathic pain through GABA release and presynaptic TRPV1 inhibition in spinal cord. Front. Mol. Neurosci. 11:248. 10.3389/fnmol.2018.00248 30065629 PMC6056657

[B54] Tang H. L. Zhang G. Ji N. N. Du L. Chen B. B. Hua R.. (2017). Toll-like receptor 4 in paraventricular nucleus mediates visceral hypersensitivity induced by maternal separation. Front. Pharmacol. 8:309. 10.3389/fphar.2017.00309 28611665 PMC5447361

[B55] Telkes I. Viswanathan A. Jimenez-Shahed J. Abosch A. Ozturk M. Gupte A.. (2018). Local field potentials of subthalamic nucleus contain electrophysiological footprints of motor subtypes of Parkinson’s disease. Proc. Natl. Acad. Sci. U S A 115, E8567–E8576. 10.1073/pnas.1810589115 30131429 PMC6130371

[B56] Trageser J. C. Keller A. (2004). Reducing the uncertainty: gating of peripheral inputs by zona incerta. J. Neurosci. 24, 8911–8915. 10.1523/jneurosci.3218-04.2004 15470158 PMC1388274

[B57] Wang X. Y. Yan W. W. Zhang X. L. Liu H. Zhang L. C. (2014). ASIC3 in the cerebrospinal fluid-contacting nucleus of brain parenchyma contributes to inflammatory pain in rats. Neurol. Res. 36, 270–275. 10.1179/1743132813y.0000000297 24512021

[B58] Watabe-Uchida M. Zhu L. Ogawa S. K. Vamanrao A. Uchida N. (2012). Whole-brain mapping of direct inputs to midbrain dopamine neurons. Neuron 74, 858–873. 10.1016/j.neuron.2012.03.017 22681690

[B59] Wei Q. Krolewski D. M. Moore S. Kumar V. Akil H. (2018). Uneven balance of power between hypothalamic peptidergic neurons in the control of feeding. Proc. Natl. Acad. Sci. U S A 115, E9489–E9498. 10.1073/pnas.1802237115 30224492 PMC6176613

[B60] Wu Y. H. Song S. Y. Liu H. Xing D. Wang X. Fei Y.. (2015). Role of adrenomedullin in the cerebrospinal fluid-contacting nucleus in the modulation of immobilization stress. Neuropeptides 51, 43–54. 10.1016/j.npep.2015.03.007 25911494

[B61] Xing D. Wu Y. Li G. Song S. Liu Y. Liu H.. (2015). Role of cerebrospinal fluid-contacting nucleus in sodium sensing and sodium appetite. Physiol. Behav. 147, 291–299. 10.1016/j.physbeh.2015.04.034 25911266

[B62] Yamanaka K. Gouraud S. S. Takagishi M. Kohsaka A. Maeda M. Waki H. (2017). Evidence for a histaminergic input from the ventral tuberomammillary nucleus to the solitary tract nucleus involved in arterial pressure regulation. Physiol. Rep. 5:e13095. 10.14814/phy2.13095 28292881 PMC5350161

[B63] Zhang G. Yu L. Chen Z. Y. Zhu J. S. Hua R. Qin X.. (2016). Activation of corticotropin-releasing factor neurons and microglia in paraventricular nucleus precipitates visceral hypersensitivity induced by colorectal distension in rats. Brain Behav. Immun. 55, 93–104. 10.1016/j.bbi.2015.12.022 26743854

[B65] Zhang N. Yang L. Guo L. Bi S. (2018). Activation of dorsomedial hypothalamic neurons promotes physical activity and decreases food intake and body weight in zucker fatty rats. Front. Mol. Neurosci. 11:179. 10.3389/fnmol.2018.00179 29896090 PMC5987017

[B64] Zhang L. C. Zeng Y. M. Ting J. Cao J. P. Wang M. S. (2003). The distributions and signaling directions of the cerebrospinal fluid contacting neurons in the parenchyma of a rat brain. Brain Res. 989, 1–8. 10.1016/s0006-8993(03)03123-8 14519505

[B66] Zhong H. Tong L. Gu N. Gao F. Lu Y. Xie R. G.. (2017). Endocannabinoid signaling in hypothalamic circuits regulates arousal from general anesthesia in mice. J. Clin. Invest. 127, 2295–2309. 10.1172/jci91038 28463228 PMC5451249

[B67] Zhou Q. Q. Chen S. S. Zhang Q. Q. Liu P. F. Fang H. Z. Yang Y.. (2017). Cerebrospinal fluid-contacting nucleus mediates nociception *via* release of fractalkine. Braz. J. Med. Biol. Res. 50:e6275. 10.1590/1414-431x20176275 28793053 PMC5572843

